# HIV clinical outcomes among people with HIV and diabetes mellitus in Kampala, Uganda; A matched retrospective cohort study

**DOI:** 10.1371/journal.pgph.0003922

**Published:** 2025-01-16

**Authors:** Rita Nakalega, Fred Collins Semitala, Edrisa Ibrahim Mutebi, Denis Mawanda, Zubair Lukyamuzi, Robert Menge, Juliet Allen Babirye, Sharon Miriam Namiiro, Cleopatra Daphne Kugonza, Nelson Mukiza, Andrew Mujugira

**Affiliations:** 1 Makerere University-Johns Hopkins University (MU-JHU) Research Collaboration, Kampala, Uganda; 2 Makerere University, Joint AIDS Program (MJAP), Kampala, Uganda; 3 Makerere University College of Health of Sciences, School of Medicine Kampala, Uganda; 4 RineCynth Advisory, Kampala, Uganda; 5 Umeå University, Umeå, Sweden; 6 The Infectious Diseases Institute, Makerere University, Kampala, Uganda; Kiruddu National Referral Hospital, UGANDA

## Abstract

Suppressive antiretroviral treatment (ART) has resulted into prolonged survival of people with HIV (PWH) in Sub-Saharan Africa (SSA) with resultant increase in the incidence of non-communicable diseases (NCD), such as diabetes mellitus (DM). However, there is a lack of data on the effect of DM on HIV-related outcomes among PWH in this setting. The study aimed to compare HIV clinical outcomes (viral load suppression, retention in care, hospitalization, tuberculosis, and mortality) between PWH with DM and those without at two large HIV clinics in Kampala, Uganda. We conducted a matched retrospective cohort study using secondary data of PWH with DM and PWH without DM from January 2020 to June 2022. We used descriptive statistics to compare baseline characteristics and a chi-square test to compare the outcomes between the HIV/DM and HIV/no DM groups. The cohort consisted of 243 PWH diagnosed with DM matched with 1221 PWH without DM. We analysed 1,469 participant records: 1,009 (68.7%) from Mulago ISS clinic and 460 (31.3%) from Kisenyi HC IV. Most study participants (63.6%) were female, and the mean age was 43 years (standard deviation [SD] 11) and 38 years (SD 10) for those with DM and without DM, respectively. PWH with DM had significantly higher odds of hospitalization (adjusted odds ratio [AOR] 4.94; 95% CI: 1.93–12.66; p = 0.001) and were less likely to be retained in care (AOR 0.12, 95% CI: 0.07–0.20 p = <0.001). There were no differences in viral load suppression, TB diagnosis, and mortality between the PWH with DM and those without DM. These findings underscore the need for integrated management approaches that address both HIV and DM to improve health outcomes for this population. Future research could also explore the causes of hospitalization and non-retention among PWH and DM.

## Introduction

Non-AIDS-related morbidity and mortality have exceeded that of AIDS-related events in the global north [1], and similar trends are being observed in sub-Saharan Africa (SSA) [2]. While all-cause mortality from opportunistic diseases is declining among PWH, there is a commensurate increase in the burden of non-communicable diseases, including diabetes mellitus (DM) [3]. This dual burden of NCDs and infectious diseases strains the health systems in low- and middle-income countries like Uganda leading to increased healthcare costs, resource challenges, and complex treatment needs [4–6]. Therefore, policymakers and researchers are increasingly recognizing the need to address NCDs [7, 8].

The increasing incidence of DM among PWH has been linked to the effects of chronic HIV infection and antiretroviral treatment (ART) [9–11]. PWH with DM experience compounded health risks because DM exacerbates renal, neurological, and cardiovascular risks due to macrovascular and microvascular DM complications [12]. DM further compromises the immune system of PWH, posing a threat to the progress reached in reducing HIV-related deaths [13]. Additionally, glycaemic control among PWH and DM is harder to achieve due to HIV infection itself and ART [14]. These DM-related adverse outcomes among PWH inform the recommendation for DM care integration in HIV clinics to allow for screening and early management, thus reducing morbidity and mortality [15, 16]. Integrated DM and HIV care reduces healthcare costs and addresses the complexities of managing the comorbidities leading to better overall health management and system efficiency [17]. Integrated DM/HIV care in study settings in Tanzania and Uganda revealed high rates of retention in care for PWH and DM and no adverse effects on viral load suppression [18]. However, there are several barriers to the implementation of integrated HIV and NCD care in Uganda that could impact the outcomes for PWH and DM [19, 20].

The DM comorbidity in HIV is prevalent in SSA, with rates as high as 26.5% [21], compared to the general population in Uganda (<1%) [22]. Yet, data regarding the clinical outcomes of PWH and DM are scarce. Available data for PWH and DM suggest that those with both HIV and DM have a higher likelihood of mortality and TB compared to PWH without DM [23, 24]. Therefore, further evaluation of DM outcomes among PWH is helpful in designing contextually relevant strategies to reduce adverse outcomes and develop appropriate models for the integration of HIV/DM care. This study, therefore, compared the HIV clinical outcomes (viral load suppression, retention in care, hospitalization, tuberculosis [TB], and mortality) among PWH with DM (PWH/DM) and those without (PWH/no DM) in Kampala, Uganda.

## Materials and methods

### Study design and setting

We conducted a matched retrospective cohort study among PWH/DM and PWH/no DM at Kisenyi Health Centre IV (HC IV) and Mulago ISS clinic in Kampala, Uganda. The Mulago and Kisenyi HIV clinics are managed and owned by the Makerere University Joint AIDS Program (MJAP) and the Kampala Capital City Authority (KCCA), respectively. Kisenyi HC IV and Mulago ISS clinics were purposefully selected because of their urban location and being the second largest and largest HIV clinics, respectively, providing HIV care in Kampala. Kisenyi HC IV serves clients from the slum areas and urban traders whereas MJAP clinic serves a diverse population of individuals. Combined, they provide vital HIV care to approximately 28,000 PWH, including 16,500 at the Mulago ISS clinic and 11,500 at Kisenyi HC IV. Treatment for HIV is available free of charge through support from the United States President’s Emergency Plan for AIDS Relief. Similarly, diagnosis and treatment for DM are provided at no cost through the public health system at Kisenyi HC IV. At the MJAP clinic, clients are referred to government or private facilities to obtain anti-diabetic medications. However, shortages of DM medications, blood glucose monitors, and test kits are common.

### DM care for PWH in Uganda

Uganda’s 2020 HIV treatment guidelines recommend screening PWH for DM risk factors and testing blood sugar levels before initiating ART if at risk. Those diagnosed with DM should adhere to national treatment protocols, including anti-diabetic medications, lifestyle modification, and referrals for specialized care, when necessary, with re-evaluation every three months. For patients starting dolutegravir (DTG), blood glucose testing is advised, especially for those at risk of hyperglycemia (e.g., age ≥45, BMI >24 kg/m^2^, history of hypertension). Quarterly monitoring during the first year of DTG treatment is also recommended [25].

### Participants and procedures

The study population was PWH newly diagnosed with DM between January 2020 and June 2022 as well as those without DM who were matched with the DM participants. PWH already diagnosed with DM prior to the specified period were excluded from the analysis. We conducted a thorough review of the medical records of PWH aged ≥18 years who were receiving healthcare from Mulago and Kisenyi HIV clinics during this period. Data were collected by trained research assistants (RAs). The RAs were comprehensively trained on the protocol, study procedures, and the standardized abstraction tool. After data clerks retrieved client files, the RAs conducted the abstraction process under the supervision of the Principal Investigator. Any records without key variables such as age, World Health Organization (WHO) clinical staging, or the ART start date were excluded from the study. Of 256 medical records retrieved, 248 were deemed suitable for analysis after data abstraction. The remaining eight records did not contain sufficient information upon enrolment and were excluded from the study. Data collected included clinical information and demographic data (age and sex) from ART/HIV care cards. These standard Ministry of Health medical charts are used for all ART clients at healthcare facilities offering ART services. If any relevant laboratory data, such as fasting or random blood sugar, HIV viral load, TB GeneXpert®, or sputum Ziehl–Neelsen results, were missing from the HIV care/ART cards, we thoroughly reviewed laboratory records to obtain the data. Subsequently, this information was extracted from the electronic medical records. Data collection took place from July to November 2023. The research team diligently reviewed all data abstraction forms to ensure high data quality standards. As an additional measure for quality control, 10% of randomly selected participant files were re-evaluated. After this thorough validation process, all data were accurately entered into EpiData (version 4.4.1).

### Statistical analysis

The primary outcome was the proportion of PWH/DM achieving HIV viral suppression (plasma HIV RNA level <1,000 copies/ml) during the follow-up period. DM was defined as 1) random venous plasma glucose ≥11.1 mmol/l or a fasting blood glucose concentration (FBG) ≥7.0 mmol/l [26] or 2) initiating oral or injected antihyperglycemic medications during the specified study period. Secondary outcomes were retention in care, hospitalization, TB diagnosis, and mortality within the 12-month follow-up period from the time of DM diagnosis for those with DM, or from the time of enrolment into the cohort for those without DM, which coincided with the matching time. Retention in care was defined as remaining alive and receiving treatment 12 months after the DM diagnosis. Hospitalization was defined as being admitted as an in-patient at least once during follow-up for any illness. A diagnosis of TB was confirmed through bacteriologically testing using a WHO-approved rapid test (i.e., Xpert MTB/RIF, lateral flow urine lipoarabinomannan assay (LF-LAM), smear microscopy, or culture) or by initiating empirical TB treatment [27]. During the one-year follow-up period from time of DM diagnosis, the total number of individuals who were documented as deceased from any cause were analysed to determine differences in mortality between the two groups (all-cause mortality was determined).

We used pair-matching to improve accuracy, minimize bias, and control for potential confounding factors [28]. PWH/DM were matched in a 1:5 ratio to PWH/no DM [using a DM prevalence of 16.6% among PWH [29]]. PWH/DM were matched to five PWH/no DM within ±1 year of age, with gender balanced and enrolment dates within ±3 months. PWH/no DM were matched only once (matching without replacement) and removed from the pool of available matches after selection. Matching without replacement preserves the independence of observations and reduces bias which increases the validity and reliability of the study results by providing more accurate comparisons between groups and thus leading to more robust conclusions [30]. The study, with 80% power, determined that with a significance level of 0.05, the minimum sample size needed was 486 participants. The proportions achieving viral suppression, experiencing hospitalization, receiving a TB diagnosis, maintaining retention in care, or death were compared between the PWH/DM and PWH/no DM groups using a Pearson chi-square test. We adjusted for confounding variables including age, sex, ART regimen, duration of treatment, and health facility at multivariable analysis for the outcomes that had significant association with DM. The level of significance was determined at 5%. Statistical analyses were conducted using Stata 16 (StataCorp, College Station, TX, USA).

### Ethics approvals

The Makerere University School of Medicine Research Ethics Committee (Mak-SOMREC-2023-599) granted ethical approval for this study. Kampala City Council Authority provided administrative clearance to conduct the study at the Kisenyi and Mulago clinics. Additionally, SOMREC waived the need for informed consent because the study involved reviewing pre-existing medical records without direct interaction with human subjects. All procedures were carried out following national guidelines and regulations.

## Results

**Fig 1:**
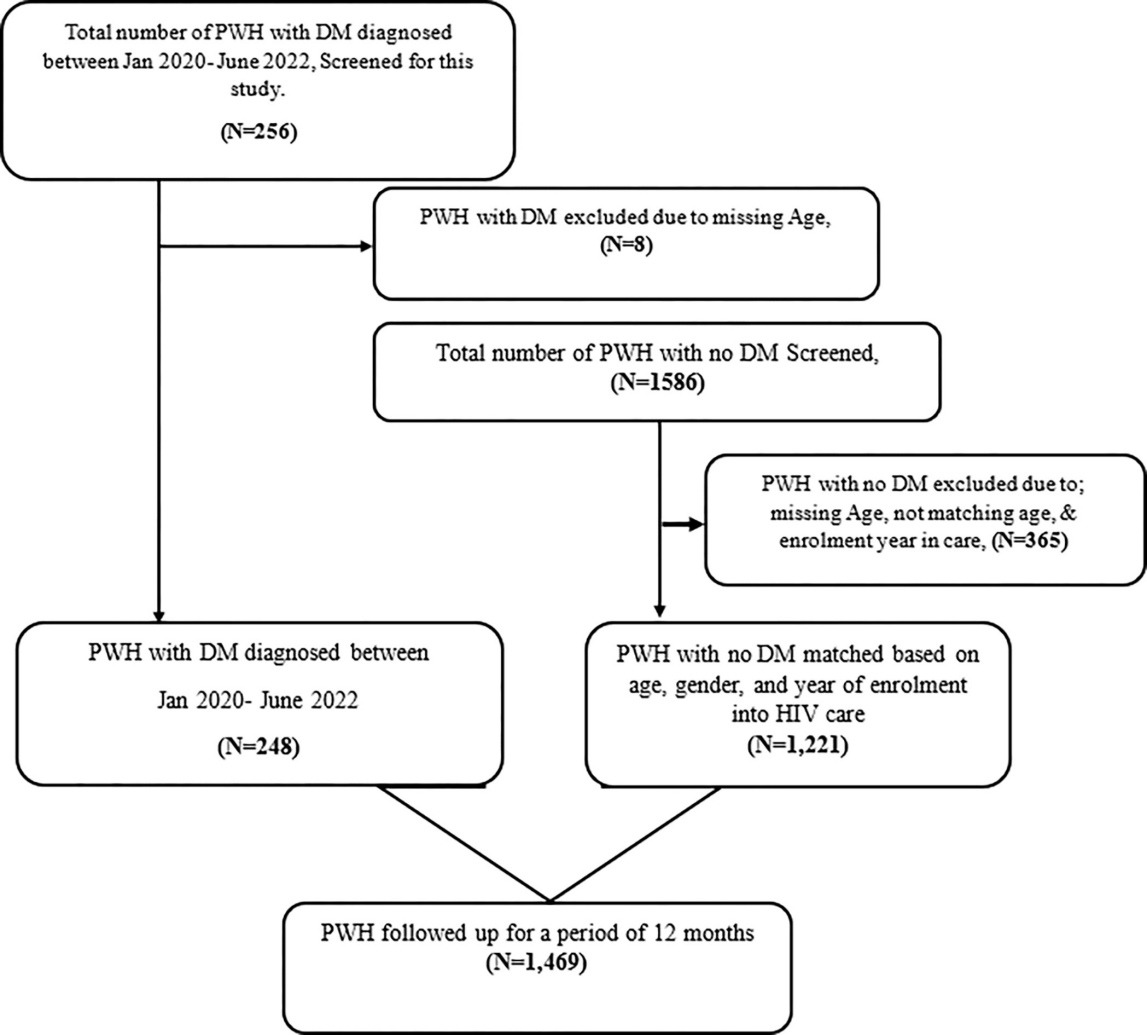
Cohort analysis flow representation.

We identified 248 PWH diagnosed with DM between January 2020 and December 2022 at Kisenyi HC IV and Mulago ISS clinic. In total, we analysed 1,469 participant records for HIV/DM and HIV/no DM: 1,009 (68.7%) were from ISS clinic Mulago, and the remaining 460 (31.3%) were from Kisenyi HC IV (Fig 1).

### Characteristics of study participants

Most study participants were female: 64.1% (159/248) among PWH/DM and 63.5% (775/1221) among PWH/no DM. Most participants were married or cohabiting: 51.4% (108/210) in the DM group and 51.7% (391/756) in the no DM group. The mean age was 42 years (SD ±11) in the DM group and 40 years (SD ±12) in the no DM group. In both groups, >65% of participants were on the tenofovir/lamivudine/ dolutegravir (TDF/3TC/DTG) regimen as their current ART regimen: 65.7% (163/248) in the DM group and 79.8% (974/1221) without DM. Furthermore, more than 70% of participants had been on ART for more than five years in both groups, [74.2% (184/248) among those with DM and 75.1% (917/1215) among those without DM] and more than 95% of the participants in each group had viral suppression at baseline. The remaining sociodemographic and clinical characteristics are presented in **Table 1**.

**Table 1:** Participant characteristics.

		DM STATUS	
Variables	Total	Has no DM	Has DM
	(n = 1469)	(n = 1221)	(n = 248)
	N (%)		
**Age in years, mean (SD)**	41 (+/-12)	40 (+/-12)	42 (+/- 11)
**Facility Name**			
Mulago ISS Clinic	1009	839 (68.7%)	170 (68.5%)
Kisenyi HC IV	460	382 (31.3%)	78 (31.5%)
**Sex at Birth**			
Female	934	775 (63.5%)	159 (64.1%)
Male	535	446 (36.5%)	89 (35.9%)
**Age of participants in complete years**			
18–35 years	575	512 (41.9%)	63 (25.4%)
36–45 years	504	414 (33.9%)	90 (36.3%)
46 years & above	390	295 (24.2%)	95 (38.2%)
**Education Level (n = 801)**			
None	36	15 (2.4%)	21 (12%)
Completed primary	350	267 (42.7%)	83 (47.4%)
Completed secondary	326	271 (43.3%)	55 (31.4%)
Completed tertiary	89	73 (11.7%)	16 (9.1%)
**Religion of participant (n = 844)**			
Born again	69	48 (7.1%)	21 (12.8%)
Catholic	393	313 (46%)	80 (48.8%)
Protestant	247	207 (30.4%)	40 (24.4%)
Moslem	134	111 (16.3%)	23 (14%)
**Others specify**	1	1 (0.1%)	0 (0%)
**Marital status of participant (n = 966)**			
Married/cohabiting	499	391 (51.7%)	108 (51.4%)
Divorced/separated	282	227 (30%)	55 (26.2%)
Single/never married	103	82 (10.8%)	21 (10%)
Others	82	56 (7.4%)	26 (12.4%)
**Occupation of participant (n = 841)**			
Employed by public Organization	105	68 (10.2%)	37 (20.9%)
Employed by private Organization	183	157 (23.6%)	26 (14.7%)
Self-employed/ Businessperson	404	319 (48%)	85 (48%)
Not working/ employed	86	69 (10.4%)	17 (9.6%)
Others	63	51 (7.7%)	12 (6.8%)
**Current ART regimen**			
TDF/3TC/DTG	1137	974 (79.8%)	163 (65.7%)
TDF/3TC/EFV	304	93 (18.3%)	80 (32.3%)
AZT/3TC/DTG	11	8 (0.7%)	3 (1.2%)
TDF/3TC/ATV/r	5	4 (0.3%)	1 (0.4%)
ABC/3TC/DTG	12	11 (0.9%)	1 (0.4%)
**WHO clinical stage**			
Stage I	878	722 (59.2%)	156 (62.9%)
Stage II	294	237 (19.4%)	57 (23.0%)
Stage III	220	186 (15.2%)	34 (13.7%)
Stage IV	77	76 (6.2%)	1 (0.4%)
**Duration on ART**			
5 years and below	368	304 (24.9%)	64 (25.8%)
Above 5 years	1101	917 (75.1%)	184 (74.2%)
Absolute CD4 cell count at enrolment (cells/mm^3^) (n = 1143)			
CD4<200	490	391 (41.6%)	99 (49%)
CD4>200	653	550 (58.4%)	103 (51%)
Baseline Viral load (VL) (n = 1441)			
Suppressed (<1000 copies/ml)	1384	1147 (96.1%)	237 (95.9%)
Non-Suppression (≥1000 copies/ml)	57	47 (3.9%)	10 (4.1%)

TDF/3TC/DTG = Tenofovir/lamivudine/ dolutegravir; TDF/3TC/EFV = Tenofovir/lamivudine/ efavirenz; AZT/3TC/DTG = Zidovudine/lamivudine/ dolutegravir; TDF/3TC/ATV/r = Tenofovir/lamivudine/ atazanavir/ritonavir; ABC/3TC/DTG = Abacavir/lamivudine/ dolutegravir

### HIV clinical outcomes

PWH with DM were significantly more likely to be hospitalised than those without DM (10.5% versus 1.4%; odds ratio [OR] 8.30, 95% confidence interval [CI]: 4.43–15.54) (**Tables 2 and 3**). This significant association remained after adjusting for HIV clinic, sex, age, completed level of education, religion, marital status, occupation, WHO clinical stage, and ART duration (adjusted odds ratio [AOR] 4.94; 95% CI: 1.93–12.66) (**Table 3**). PWH with DM were notably less likely to be retained in care compared to those with only HIV (37.1% versus 18.3%; p = 0.0001. The odds of not remaining engaged in care during the 12-month study period were nearly three times higher for PWH with DM (OR 0.38; 95% CI: 0.28–0.51). Additionally, after adjusting for age, sex, education level, religion, occupation, and CD4 cell count at enrolment, this association changed significantly, indicating a positive confounding effect (adjusted odds ratio [AOR] 0.12; 95% CI: 0.74–0.20) (**Table 3**). Notably, there was no significant difference in viral load suppression change between PWH with DM and those without DM (p = 0.242). At the end of the follow-up period, 5.9% of participants without DM and 3.2% of those with DM became non-suppressed. Meanwhile, 90.9% of participants without DM and 93.1% of those with DM remained with the same viral load status from baseline to follow-up. Lastly, 3.2% of the participants without DM and 3.6% of those with DM showed improved viral load suppression by the end of the follow-up period. Similarly, there was no significant difference in the likelihood of being diagnosed with TB between PWH with DM (6.1%) and those without DM (4.7%) (p = 0.36) (**Table 2**). Lastly, the two groups had no disparity in mortality (2% versus 3.2%, p = 0.32).

**Table 2:** Bivariate analysis of the association between DM status and outcomes among PWH over the 12-month follow-up period.

Association between DM status and outcomes among PWH over the 12-month follow up period, n = 1469	DM Status			Pearson Chi2
				Chi-square value
	No DM (%)	Has DM (%)	Total (%)	P-value
**Hospitalized**				
Yes	17 (1.4)	26 (10.5)	43 (2.9)	59.9
No	1204 (98.6)	222 (89.5)	1426 (97.1)	**p < 0.001** ***
**Participant still in care after 12 months**				
Yes	997 (81.7)	156 (62.9)	1153 (78.5)	42.9
No	224 (18.3)	92 (37.1)	316 (21.5)	**p < 0.001** ***
**Diagnosed with Tuberculosis (TB)**				
Yes	57 (4.7)	15 (6.1)	72 (4.9)	0.84
No	1164 (95.3)	233 (93.9)	1397 (95.1)	p = 0.36
**Change in Viral load status**				
Worsened (increased to >1000 copies/ml	70 (5.9)	8 (3.2)	78 (5.4)	
No change	1086 (90.9)	230 (93.1)	1316 (91.3)	
Improved (declined to <1000 copies/ml	38 (3.2)	9 (3.6)	47 (3.3)	P = 0.243
**Participant died**				
**Yes**	39 (3.2)	5 (2.0)	44 (3.0)	0.98
**No**	1182 (96.8)	243 (98)	1425 (97.0)	p = 0.32

**Table 3:** Multivariate analysis of associations between DM status and HIV outcomes among PWH over a 12-month follow-up period.

Variables	Total	Hospitalized (%)	Not Hospitalized (%)	P-value	COR	P-value	AOR
Has no DM	1221	17 (1.4)	1204 (98.6)		1		1
Has DM	248	26 (10.5)	222 (89.5)	**p<0.001**	**8.30 (4.43–15.54)**	**0.001**	**4.94 (1.93–12.66)**
**Variables**	**Total (%)**	**Retained (%)**	**Not Retained (%)**	**p-value**	COR (95% CI)	p-value	AOR (95% CI)
Has no DM	1221 (83.1)	224 (18.3)	997 (81.7)		1	.	1
Has DM	248 (16.9)	92 (37.1)	156 (81.7)	**p<0.001**	**0.38 (0.28–0.51)**	**p<0.001**	**0.12 (0.07–0.20)**

*DM; Diabetes mellitus, COR; crude odds ratio, AOR; Adjusted odds ratio

## Discussion

This retrospective cohort analysis, conducted at two large HIV clinics in Kampala, Uganda, examined the clinical outcomes of approximately 250 PWH. The results showed that PWH with DM had higher odds of hospitalization and lower retention in care over the 12-month study period than those without DM. However, there were no significant differences in the proportions of individuals achieving viral load suppression, being diagnosed with tuberculosis (TB), or experiencing mortality between the two groups during this timeframe.

We found that hospitalization rates were significantly higher in the PWH/DM group during the 12-month observation period. PWH/DM without good glycaemic control are more susceptible to microvascular and macrovascular complications, such as coronary artery disease, stroke, peripheral vascular disease, retinopathy, and chronic kidney disease. These complications can lead to hospitalizations [12]. Factors that hinder treatment adherence, such as lack of access to medications, can further increase the risk of hospitalization in individuals with comorbid DM [31]. A study in Ethiopia similarly reported increased odds of hospitalization among individuals living with both HIV and diabetes [32]. Increased hospitalization rates not only exacerbate the strain on healthcare systems in sub-Saharan Africa [33] but also negatively impact the overall health of PWH leading to increased morbidity and mortality in this population [34]. On the other hand, retention in care was significantly lower among PWH with comorbid DM compared to those without. This low retention in care for PWH with DM has significant implications on their ability to adhere to anti-diabetic medication regimens, monitor their glycaemic control levels regularly, and identify potential complications at an early stage [35–37]. Previous research has indicated no significant differences in retention rates for PWH with DM than those without DM when studied within modelled communities in Uganda and Tanzania [18]. However, studies conducted in Sub-Saharan Africa have shown lower retention rates after a diagnosis of DM or hypertension in the general population in comparison to PWH and DM [37–39]. Barriers that may contribute to non-retention include undocumented mortality, interruptions in drug supplies, high costs associated with accessing care, and inadequate provision of health education in low-resource settings like Uganda [40]. Addressing these issues could improve health outcomes for PWH with DM. A retrospective cohort study conducted in the United States found that PWH with at least one non-HIV-related comorbidity had better retention rates than those without comorbidities [41]. Specifically, PWH with DM were twice as likely to remain engaged in care. This trend is linked to the integrated healthcare systems, comprehensive care models, and robust patient support programs, which enhance access to resources and health insurance coverage. These factors collectively contribute to better overall management and retention in care, a pattern also seen in other developed nations [42, 43].

There were no differences in viral load suppression between the two groups. Over 65% of participants in both cohorts were on dolutegravir (DTG), an effective ART regimen known for rapid and sustained viral suppression [44, 45]. The combination of a DTG-based regimen expanded viral load testing, and adherence counselling may explain the lack of difference in viral suppression between the cohorts [46]. Similarly, studies in Uganda and South Africa found no significant difference in viral suppression between people with HIV who had hypertension or other non-communicable diseases (NCDs) and those without these comorbidities [47, 48]. While our study did not find any notable contrast in the likelihood of being diagnosed with TB between PWH with or without DM, several studies have shown an association between DM and TB among PWH [23, 49]. The association between DM and increased susceptibility to TB is believed to result from hyperglycaemia, insulin resistance, and their indirect effects on macrophage and lymphocyte function [50]. Our one-year retrospective analysis may have missed some TB cases due to limited documentation and suboptimal case-finding efforts. Additionally, challenges within the healthcare system, such as inadequate active TB screening, likely contributed to underreporting of TB cases [51].

Although our study did not find statistically significant differences in mortality rates between PWH with and without DM probably due to a limited follow-up period, other research has suggested a potential link between DM and mortality among PWH. One cohort study followed 4,871 adults with HIV for ten years and found that death rates were significantly higher (RR 1.74; p< 0.001) among those with DM compared to those without DM, with 1,192 deaths recorded [34]. A separate cohort study in Thailand also found that PWH diagnosed with DM had a higher risk of death compared to those without DM after eight years of follow-up. This finding aligns with previous research conducted in the same country [52, 53].

### Strengths and limitations

Our study has several strengths, including a cohort design that assessed temporal relationships between DM and hospitalization and retention in care. Additionally, our large sample size of approximately 1500 PWH provided robust data for analysis. By evaluating multiple outcomes, such as viral load suppression, TB, mortality, hospitalization, and retention in care following DM diagnosis, our study sheds light on the impact of DM on these outcomes among PWH. Furthermore, given the urban setting of our study and large population of PWH served by both clinics (> 2800 PWH), the results can be considered representative of HIV clinics in similar settings. However, a limitation of the study is its reliance on routine clinic records, which had missing data. To address this issue, we conducted a complete case analysis, excluding cases with missing values. This approach may have also reduced the statistical power of the test, making it less likely to detect a true association.

## Conclusions

This study highlights the significant impact of DM comorbidity on clinical outcomes among PWH in Uganda. Over 12 months, PWH with DM had significantly higher odds of hospitalization and were less likely to remain in care than PWH without DM. These findings highlight the critical need for integrated management strategies in managing PWH with DM, given the higher risk of complications that lead to hospitalizations and poor retention in care. However, the higher rates of hospitalization and lower retention in care observed among PWH with DM highlight the need for tailored interventions to manage DM-related complications and ensure continued engagement in care. Future research should focus on developing integrated care models for effectively managing PWH with comorbid DM and investigating the factors contributing to hospitalization and non-retention among this population subgroup.

## Supporting information

S1 Data HIV clinical outcome in PWH and DM dataset. (XLSX)
